# Supplementary material for: Evidence supported by Mendelian randomization: impact on inflammatory factors in knee osteoarthritis
Source: Front Med (Lausanne). 2024 May 28;11:1382836. doi: 10.3389/fmed.2024.1382836 (PMC11165061; doi:10.3389/fmed.2024.1382836)
Supplement: Supplementary file 1 [file Data_Sheet_1.ZIP › data/GCST90274788_5e6/788.forest.pdf]

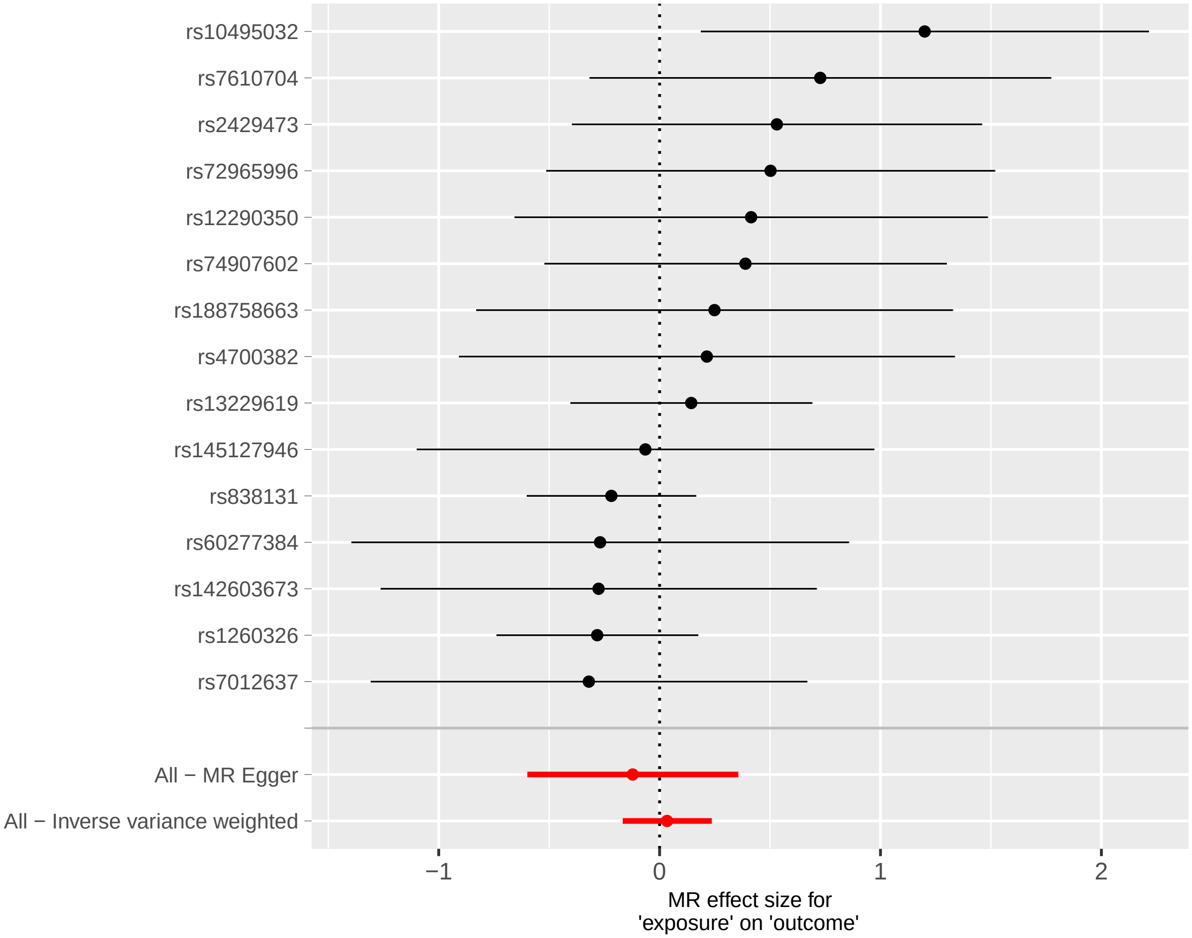
rs10495032
rs7610704
rs2429473
rs72965996
rs12290350
rs74907602
rs188758663
rs4700382
rs13229619
rs145127946
rs838131
rs60277384
rs142603673
rs1260326
rs7012637
All − MR Egger
All − Inverse variance weighted
−1
0
1
2
MR effect size for
'exposure' on 'outcome'
